# The role of challenging exercise in improving behavioral dysfunction in a mouse model of Parkinson’s disease

**DOI:** 10.3389/fnbeh.2026.1887753

**Published:** 2026-09-17

**Authors:** Yuan Zeng, Ping Chen, Quan Yang, Bing Liu, Liuting Huang, Kai Zhang, Shumin Liu

**Affiliations:** 1 College of Sports Science, Jishou University, Hunan, China; 2 College of General Education, Tumushuk Vocational and Technical College, Tumushuk, China

**Keywords:** behavioral dysfunction, brain-derived neurotrophic factor, challenging exercise, Parkinson’s disease, tyrosine receptor kinase B

## Abstract

**Objective:**

To investigate whether accelerated rotarod exercise preconditioning provides greater neuroprotection than constant-speed training in a 6-OHDA mouse model of Parkinson’s disease (PD).

**Methods:**

Male C57BL/6 mice were assigned to four groups: sham-operated sedentary (Con), PD sedentary (PD), constant-speed exercise + PD (Gen+PD, 10 rpm), and accelerated exercise + PD (Acc+PD, 5–40 rpm). Both exercise groups completed 4 weeks of rotarod training (240 s/session, 6 sessions/day, 5 days/week) before unilateral intrastriatal 6-OHDA injection. Con mice received vehicle at identical coordinates. Motor function was assessed by APO-induced rotation, negative geotaxis, pole, and open field tests. Striatal dopamine (DA) was quantified by ELISA; BDNF, TrkB, and TH expression were measured at the mRNA (RT-qPCR) and protein (Western blot) levels in the striatum and substantia nigra.

**Results:**

Relative to the PD group, Acc+PD mice showed significant reductions in negative geotaxis turning latency, T-turn, and T-total (all *P* < 0.01), and significant increases in total locomotor distance, mean speed, and central zone dwell time (all *P* < 0.01). Striatal DA content was markedly elevated in the Acc+PD group (*P* < 0.01). BDNF and TrkB mRNA were significantly upregulated in both the striatum and substantia nigra (*P* < 0.01), and TH, BDNF, and TrkB protein levels in the substantia nigra were substantially increased (*P* < 0.01). Improvements in the Gen+PD group were more modest and restricted in scope. Across all indices, the Acc+PD group consistently outperformed the Gen+PD group.

**Conclusion:**

Four weeks of acceleration-based rotarod preconditioning substantially delayed behavioral deficits in 6-OHDA-lesioned mice, accompanied by greater preservation of striatal DA and broader upregulation of TH, BDNF, and TrkB compared with constant-speed training. These findings identify exercise intensity as a critical determinant of preconditioning efficacy and support accelerated rotarod training as a more effective neuroprotective strategy in this PD model.

## 1 Introduction

Parkinson’s disease (PD) is the second most common disabling neurodegenerative disorder after Alzheimer’s disease (AD) (Poewe et al., 2017). The primary pathological features of PD include progressive loss of dopamine (DA) neurons in the substantia nigra pars compacta (SNpc) and a significant reduction in striatal DA levels (Chen et al., 2017; Zhao et al., 2020; Shi et al., 2020; Hu et al., 2021). Clinically, PD is characterized by motor symptoms such as bradykinesia, resting tremor, muscle rigidity, and postural instability (Simon et al., 2020), which severely impair patients’ activities of daily living and diminish their quality of life (Zhang et al., 2022). As early as 1992, researchers observed that individuals who engaged in regular physical activity during early adulthood had a significantly lower lifetime risk of developing PD (Sasco et al., 1992). Subsequent large-scale epidemiological studies have further confirmed the beneficial effects of exercise on PD, and accumulating evidence indicates that exercise can substantially attenuate the risk of neurological deterioration. Among these, structured exercise therapy—spanning aerobic training, resistance exercise, and balance-focused regimens—has demonstrated measurable benefits on motor symptoms including gait speed, postural stability, and fall frequency in both clinical trials and community-based programs. However, randomized controlled trials directly comparing the efficacy of different exercise types on PD-related motor performance remain scarce. Against this backdrop, identifying exercise protocols that maximize neuroprotective and functional benefits—while remaining clinically implementable—represents a pressing research priority in PD management. Allen et al. highlighted in a meta-analysis that current exercise interventions for PD often lack sufficient challenge and intensity (Allen et al., 2011). They emphasized the importance of increasing both the difficulty and the total volume of training in future programs. Specifically, higher-dose training and highly challenging balance exercises—such as those involving weight shifting, reduced base of support, and minimized upper limb assistance—were suggested to produce greater effects on balance-related performance and may be necessary for reducing fall risk. Studies by Ahlskog and Petzinger et al. further demonstrated that high-intensity, challenging, and cognitively demanding exercise can induce neuroplasticity (Ahlskog, 2011; Petzinger et al., 2013). In this context, it is important to note that the term “high-challenge training” as used across the literature encompasses multiple distinct dimensions: (i) physical intensity, referring to parameters such as speed, resistance, and duration of exercise; (ii) task complexity, reflecting the progressive or unpredictable demands of the motor task itself, such as accelerating speeds or variable surface conditions; and (iii) motor-cognitive demand, involving concurrent engagement of cognitive resources during motor execution, such as dual-task paradigms or real-time postural adjustment under changing conditions. While these dimensions frequently overlap, distinguishing among them is essential for defining exercise protocols with precision and for interpreting the specificity of their neurobiological effects. The accelerating rotarod paradigm employed in the present study primarily engages task complexity and sensorimotor adaptability—requiring mice to continuously adjust their gait and postural strategy in response to progressively increasing rotational speed—rather than simply increasing aerobic workload at a fixed intensity. Numerous experimental studies (Leavy et al., 2020; Leavy et al., 2021; Joseph et al., 2018) and meta-analyses (Farlie et al., 2016; Malmir and Ashrafganjooie, 2023) have shown that high-challenge training leads to significant short-term improvements in balance and gait impairments in PD patients. Despite these promising findings, the neurobiological pathways through which challenging exercise confers motor benefits remain poorly defined. In particular, whether high-challenge training protocols can extend their benefits to PD-related deficits beyond balance and gait—such as spontaneous locomotion, anxiety-like behavior, and dopaminergic signaling—has not been directly tested in preclinical models.

Translating these clinical observations into mechanistic understanding requires the use of well-characterized animal models of PD. The unilateral 6-hydroxydopamine (6-OHDA) lesion model is one of the most widely used and best-validated rodent models for studying the progressive degeneration of nigrostriatal dopaminergic neurons (Simola et al., 2007). Intracerebral injection of 6-OHDA selectively destroys catecholaminergic neurons through oxidative stress-mediated mechanisms, producing quantifiable motor deficits and a reproducible pattern of DA depletion that closely recapitulates key features of human PD pathology (Barata-Antunes et al., 2020). Importantly, subsequent neurodegenerative processes in this model involve not only dopaminergic cell loss but also neuroinflammatory glial responses and apoptotic cascades that contribute to the progressive nature of the lesion (Freire et al., 2025). Using such toxin-based models, a growing body of preclinical evidence has demonstrated that exercise training can attenuate dopaminergic neurodegeneration, enhance synaptic plasticity, and preserve nigrostriatal pathway integrity. For example, treadmill exercise has been shown to protect tyrosine hydroxylase (TH)-positive neurons in the SNpc, restore striatal DA concentrations, and upregulate expression of synaptic markers in both 6-OHDA and MPTP mouse models (Tajiri et al., 2010; Petzinger et al., 2013). However, the majority of these studies have employed constant-speed or fixed-intensity protocols, leaving unanswered whether exercise paradigms that incorporate progressive task complexity—more closely mimicking the “high-challenge” training advocated in clinical literature—confer additional neuroprotective benefits at the molecular level.

Research indicates that PD neurodegeneration is closely associated with mitochondrial dysfunction, oxidative damage, and apoptosis (Gao et al., 2022). Neurotrophic factors are recognized as key modulators of neuronal survival, acting in part through the regulation of mitochondrial biogenesis and the suppression of oxidative stress-driven apoptosis (Jin, 2020). Collectively, these observations position neurotrophic factor signaling as a mechanistically tractable target in the context of PD neurodegeneration. It has been well established that exercise training elevates brain-derived neurotrophic factor (BDNF) levels and improves brain function (Kaagman et al., 2024). Exercise further upregulates the mRNA expression of BDNF, its receptor TrkB, the transcription factor CREB, and synapsin I, collectively enhancing synaptic plasticity and neurotransmitter release (Jang et al., 2017; Wu et al., 2011; Małczyńska et al., 2019). Building on this evidence, the present study tested whether acceleration-based challenging rotarod training—relative to constant-speed conventional exercise—more effectively preserves nigrostriatal DA integrity, attenuates motor deterioration, and amplifies BDNF/TrkB signaling in a 6-OHDA mouse model of PD.

## 2 Experimental subjects and methods

### 2.1 Experimental subjects and grouping

Male C57BL/6 mice, weighing 20–24 g (8–9 weeks old), were purchased from Hunan Silaike Jingda Experimental Animal Co., Ltd. (Animal Production License No.: SCXK (Xiang) 2021-0002). All mice were individually housed at 22 ± 1 °C with 60–65% relative humidity under a 12:12 h light-dark cycle, with *ad libitum* access to food and water. All animals were allowed 1 week of acclimatization to the housing environment and familiarization with the rotarod apparatus before experimental procedures commenced. Mice were then randomly assigned to one of four groups (*n* = 12 per group): Sham-operated Sedentary group (Con), PD Sedentary group (PD), General Exercise Preconditioning + PD group (Gen+PD), and Accelerated/Challenging Exercise Preconditioning + PD group (Acc+PD).

All 48 mice completed the full experimental protocol with no attrition or exclusion. All animals (*n* = 12 per group) underwent behavioral assessments. Following behavioral testing, animals within each group were randomly subdivided into two cohorts for downstream tissue analyses: Cohort 1 (*n* = 6 per group) was used for striatal dopamine quantification by ELISA and immunohistochemical analysis of TH in the substantia nigra; Cohort 2 (*n* = 6 per group) was used for RT-qPCR analysis of BDNF and TrkB mRNA in both the striatum and substantia nigra, as well as Western blot analysis of TH, BDNF, and TrkB protein in the substantia nigra. Because the mouse substantia nigra is extremely small and the tissue yield from a single animal was insufficient to simultaneously perform both RT-qPCR and Western blot with adequate input material, the substantia nigra tissue from the 6 animals per group in Cohort 2 was processed as three biological replicate pools (two mice per pool), from which RNA and protein were simultaneously extracted using TRIzol reagent (dual-extraction method).

### 2.2 Training protocol

Before random grouping, all mice were trained on an accelerating rotarod (4–40 rpm) for 300 s per session, with at least 1-hour rest intervals, twice daily for 5 consecutive days. Following group assignment, mice in the Gen+PD group performed rotarod exercise at a constant speed (10 rpm), while mice in the Acc+PD group performed rotarod exercise under an accelerating speed protocol (5–40 rpm). Each session lasted 240 s, separated by 240 s inter-session rest intervals, with 6 sessions completed per day across 5 consecutive training days per week over a 4-week period. Mice in the Con and PD groups were placed on the stationary rotarod apparatus for equivalent durations without active running, serving as sedentary controls (Zhang et al., 2024).

### 2.3 Establishment of PD model mice

Unilateral intrastriatal 6-OHDA injections were performed under deep anesthesia according to Wang et al. (Wang et al., 2022). With reference to a mouse brain stereotaxic atlas, the right striatum was targeted at the following coordinates: AP: +0.5 mm, ML: +2.0 mm, with injections delivered at two depths (DV: -3.8 mm and -3.0 mm; 1.5 μL per site). 6-OHDA was dissolved in 0.9% saline containing 0.02% ascorbic acid at a concentration of 2 μg/μL. Using a 10 μL microsyringe, the solution was infused at a constant rate of 0.5 μL/min. Following each injection, the needle was left in place for 5 min before being slowly withdrawn at a rate of 0.5 μL/min. Con group mice underwent identical surgical procedures, with the vehicle solution (0.9% saline + 0.02% ascorbic acid) substituted for 6-OHDA at the same stereotaxic coordinates (Figure 1).

**FIGURE 1:**
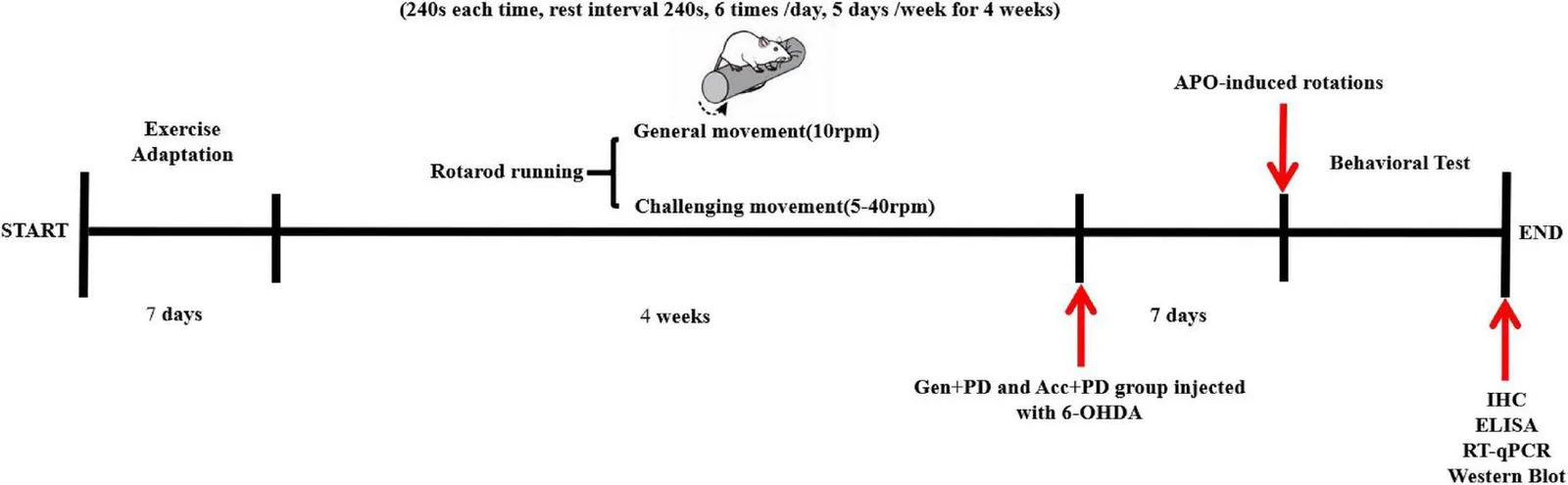
Experimental procedures.

### 2.4 APO-induced rotation test

Seven days after 6-OHDA injection, apomorphine (APO)-induced rotation testing was performed. Mice received a subcutaneous injection of APO solution (0.125 g/L, 0.35 ml/kg). After a 5-min latency period, the number of complete full-body rotations (360°) within 30 min was recorded. A net difference exceeding 120 contralateral rotations (toward the intact left side versus the lesioned right side) within 30 min was used as the criterion for successful PD model induction (Wang et al., 2025). Only mice meeting this criterion were included in subsequent experimental procedures.

### 2.5 Behavioral tests

#### 2.5.1 Negative geotaxis test

The negative geotaxis test was used to evaluate balance ability in mice (Thiessen and Lindzey, 1967). The apparatus consisted of a 45° inclined acrylic platform. Mice were placed in a head-down position, and the latency to rotate 180° from head-down to head-up was recorded as the negative geotaxis time (seconds). The platform surface was wiped with 70% ethanol between trials to eliminate olfactory cues. Each mouse was manually restrained in a head-down position at the top of the incline for 5 s prior to release. Three trials were conducted per animal, and the mean latency across trials was used for statistical analysis.

#### 2.5.2 Pole test

The pole test was used to evaluate motor coordination in mice (Tajiri et al., 2010). The pole (50 cm height, 1.0 cm diameter) was wrapped with medical gauze to improve grip, and a wooden ball was affixed to the top to discourage prolonged stationary positioning. Mice were placed head-up at the top of the pole, and two parameters were recorded: the latency to turn downward (T-turn) and the total time to descend to the base (T-total). Each animal completed three trials, and the mean T-turn and T-total values were used for subsequent analysis.

#### 2.5.3 Open field test

The open field test (OFT) was used to evaluate spontaneous locomotor activity in mice. Locomotor activity was captured by an overhead SONY camera and analyzed using ANY-Maze video tracking software (Stoelting) over a 3-min free-exploration period. Raw data were subsequently processed with SMART 3.0 software to quantify total distance traveled, mean locomotor speed, and time spent in the central zone. To minimize novelty-induced stress responses, mice were introduced to the arena once daily for three consecutive days prior to testing.

Behavioral tests were conducted over three consecutive days in a fixed order (Day 1: negative geotaxis test; Day 2: pole test; Day 3: open field test) to avoid carry-over effects of fatigue or stress between tests. The testing order was identical across all four groups.

### 2.6 Immunohistochemical staining for TH

For Cohort 1 mice, 24 h after the completion of behavioral tests, mice were deeply anesthetized and then decapitated. The right striatum was rapidly dissected on ice and processed for ELISA (see section 2.7). The remaining brain tissue was immediately immersed in 4% paraformaldehyde at 4 °C for 24–48 h for post-fixation. Following fixation, brains were dehydrated through a graded ethanol series, cleared in xylene, and embedded in paraffin, and trimmed to expose the region of interest. The positions of the striatum and substantia nigra were identified according to the mouse brain stereotaxic atlas (Paxinos and Franklin, 2012). Coronal sections were cut continuously, with every third section collected for staining. Selected sections were washed in 0.01 M PBS (pH 7.4), permeabilized with 0.3% Triton X-100 in PBS for 30 min at room temperature, and treated with 3% H_2_O_2_ for 10 min to quench endogenous peroxidase activity, followed by additional PBS washes. Sections were then blocked with 5% normal goat serum in PBS for 1 h at room temperature and incubated overnight with mouse anti-tyrosine hydroxylase (TH) monoclonal antibody (1:3,000; Sigma, United States). After three PBS washes, sections were incubated with biotinylated rabbit anti-mouse secondary antibody for 1 h at room temperature, followed by avidin-biotin-peroxidase complex (ABC-Elite kit, Vector Laboratories, United States) for 1 h. After three additional PBS washes, immunoreactivity was visualized with DAB solution for 10–20 s. Images were acquired using an Olympus-DP72 microscope, and mean optical density of TH-immunopositive cells was quantified with Image-Pro Plus 6.0 software as an index of dopaminergic neuron loss in the nigrostriatal pathway.

### 2.7 Enzyme-linked immunosorbent assay (ELISA) for striatal DA concentration

As described in section 1.6, Cohort 1 mice were deeply anesthetized and decapitated 24 h after behavioral testing. The right striatum was rapidly dissected on ice, snap-frozen, and stored at -80°C in sterile microcentrifuge tubes. Striatal DA content was quantified using a commercially available ELISA kit (one-step sandwich method), with all procedures carried out in strict accordance with the manufacturer’s instructions. Striatal tissue was weighed and homogenized in ice-cold 0.9% saline at a tissue-to-saline ratio of 1:9 (w/v). The homogenate was centrifuged at 3,000 rpm for 10 min at 4°C, and the resulting supernatant was collected and stored at -80°C until analysis. Supernatants were diluted as required prior to DA detection. Absorbance was measured at 450 nm using a microplate reader, and DA concentrations were interpolated from a standard curve generated by linear regression of OD values against known standard concentrations. Each sample was measured in triplicate across three independent experiments.

Striatal tissue was weighed and homogenized in ice-cold 0.9% saline at a tissue-to-saline ratio of 1:9 (w/v), ensuring consistent normalization of DA concentrations to tissue mass across all samples. Each sample was measured in triplicate across three independent experiments. The intra-assay and inter-assay coefficients of variation were < 10 and < 15%, respectively, as specified by the kit manufacturer.

### 2.8 Real-time quantitative PCR (RT-qPCR) for BDNF and TrkB mRNA expression in striatum and substantia nigra

For Cohort 2 mice, 24 h after the completion of behavioral tests, mice were deeply anesthetized and decapitated. The right striatum and substantia nigra were rapidly dissected on ice. Striatal tissue was homogenized in TRIzol reagent (Invitrogen, United States) for RNA extraction. Substantia nigra tissue was also homogenized in TRIzol reagent; RNA was isolated from the aqueous phase according to the manufacturer’s instructions, while protein was simultaneously recovered from the organic/interphase for Western blot analysis (see section 2.9). This dual-extraction strategy enabled the acquisition of both RNA and protein from the same substantia nigra sample, which was necessary given the limited tissue mass of this region in mice. RNA integrity and yield were assessed spectrophotometrically; only samples with A260/A280 ratios between 1.8 and 2.2 were used for downstream analysis. First-strand cDNA was synthesized from total RNA using a commercial cDNA synthesis kit per the manufacturer’s protocol. qPCR was performed using SYBR^®^ Green Realtime PCR Master Mix under the following thermal cycling conditions: initial denaturation at 94°C for 30 s, then 40 cycles of 94°C for 5 s and 55°C for 30 s (combined annealing/extension). GAPDH served as the internal reference gene, and relative mRNA expression was calculated using the 2-ΔΔCt method. All primers were custom-synthesized by Aoke (Wuhan) Biotechnology Co., Ltd.; sequences are provided in Table 1.

**TABLE 1:** Primer sequences.

Gene	Primers sequence	Length/ bp
BDNF	F:5’-TAGGCAGAATGAGCAATGTC-3’	178
	R:5’-CCCAAGAGGTAAAGTGTAGAAG-3’	
TrkB	F:5’-TGCTCAAGTTGGCGAGACAT-3’	304
	R:5’-GTCCCAGGAGTTCAGCTCAC-3’	
GAPDH	F:5’-AGGAAATGATGACCTCCTGAACT-3’	79
	R:5’-TGTTTTTGTAAGTATCTTGGTGCCT-3’	

Calculation method: The Ct values in the results are analyzed using the 2^ΔΔCt method, and the relative expression levels of the genes are calculated.

### 2.9 Western blot analysis of TH, BDNF, and TrkB protein expression in the substantia nigra

As described in section 2.8, for Cohort 2 mice, protein was recovered from the organic phase and interphase of TRIzol-homogenized substantia nigra samples following RNA extraction from the aqueous phase. Protein pellets were washed sequentially with 0.3 M guanidine hydrochloride in 95% ethanol and acetone, air-dried, and solubilized in 1% SDS lysis buffer. Protein concentrations were determined by BCA assay. Samples were then mixed with 5 × SDS loading buffer, denatured at 100°C for 5 min, and stored at -80°C until use. For Western blotting, 30 μg of total protein per sample was resolved by SDS-PAGE and transferred onto a PVDF membrane. Membranes were blocked with 5% non-fat dry milk in TBST for 90 min at room temperature with gentle agitation. Following blocking, membranes were incubated with primary antibodies at 4°C overnight, washed three times with TBST, and then incubated with HRP-conjugated secondary antibodies at room temperature for 90 min, followed by three additional TBST washes. Immunoreactive bands were visualized by applying enhanced chemiluminescence (ECL) substrate to the membrane, with signals captured on X-ray film. GAPDH was used as a loading control. Band intensities were quantified using ImageJ software, and protein expression was normalized to the corresponding GAPDH signal within each lane.

### 2.10 Statistical analysis

Statistical analyses were performed using SPSS 26.0 software, and graphs were generated with GraphPad Prism 9.5. Measurement data are expressed as mean ± standard deviation (mean ± SD). For indicators with larger sample sizes (*n* ≥ 6 per group), the Shapiro-Wilk test and Levene’s test were used to assess normality and homogeneity of variance, respectively, and all datasets met these assumptions. For Western blot and RT-qPCR data (*n* = 3 biological replicate pools per group), parametric tests were applied based on the biological prior knowledge of such molecular indicators and common practice in the field. Comparisons among three or more groups were performed using one-way analysis of variance (ANOVA), followed by Tukey’s HSD post hoc test for pairwise intergroup comparisons. A *P* value < 0.05 was considered statistically significant.

## 3 Results

### 3.1 Apomorphine-induced rotation test and TH immunohistochemistry

#### 3.1.1 Apomorphine-induced rotation test

APO-induced rotation behavior was evaluated on post-injection day 7 to confirm successful unilateral dopaminergic lesioning. No animals were excluded based on this criterion in the present study, as all lesioned mice surpassed the 120-rotation threshold.

The differences in apomorphine-induced rotation counts among the groups were statistically significant [one-way ANOVA, *F*(3, 20) = 80.37, *P* < 0.01, η^2^*p* = 0.923). Post-hoc analysis revealed that the rotation counts in the PD group were significantly higher than those in the control group (582 ± 129 vs. 3 ± 2 turns/30 min, *P* < 0.01, Cohen’s *d* = 6.35); the rotation counts in the Acc+PD group were significantly lower than those in the PD group (247 ± 18 vs. 582 ± 129 turns/30 min, *P* < 0.01, Cohen’s *d* = 3.64); and the rotation counts in the Acc+PD group were significantly lower than those in the Gen+PD group (247 ± 18 vs. 331 ± 14 turns/30 min, *P* < 0.05, Cohen’s *d* = 5.22) (Figure 2).

**FIGURE 2:**
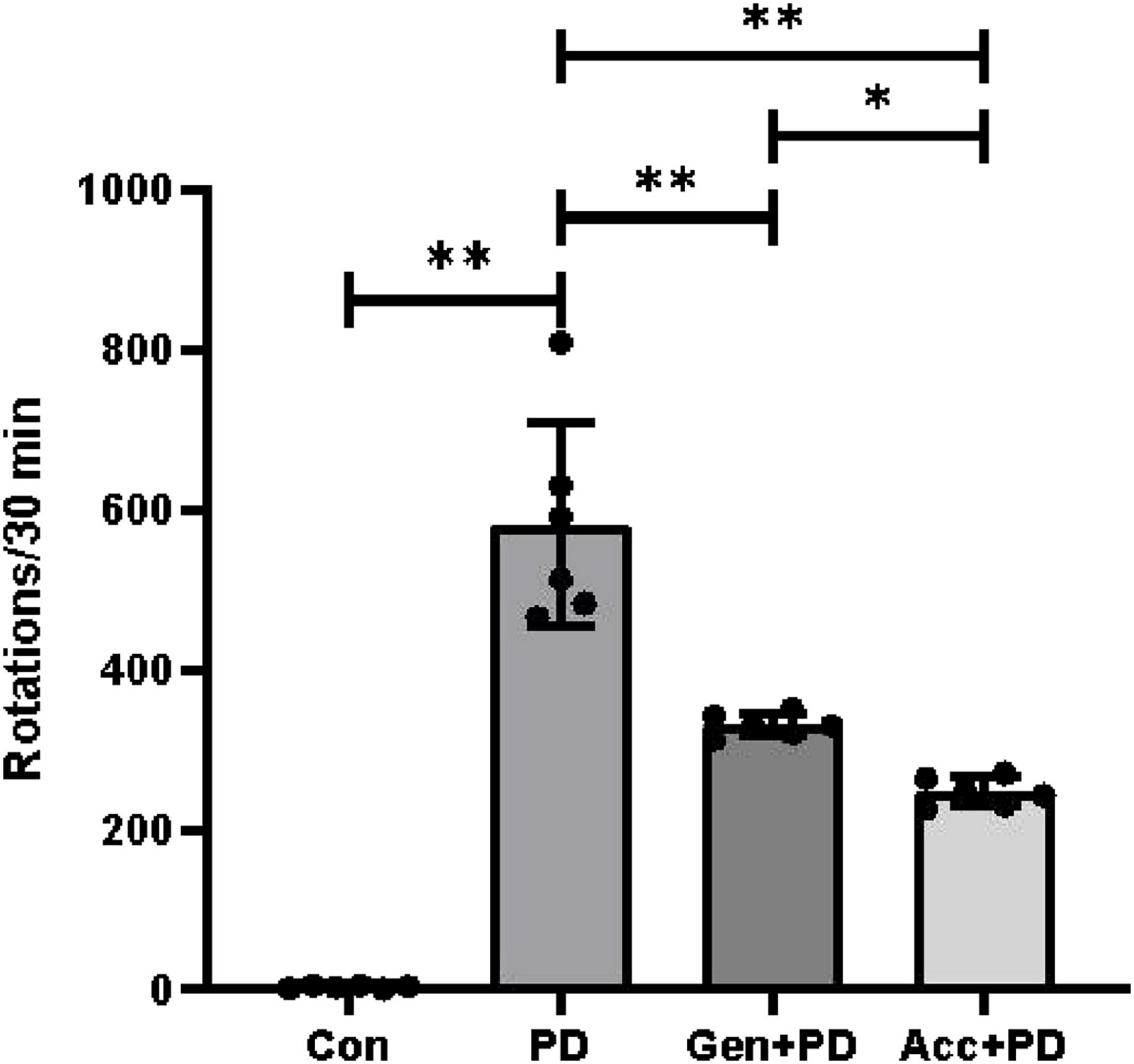
Comparisons of apomorphine-induced rotation behavior. *P < 0.05, **P < 0.01.

#### 3.1.2 TH immunohistochemistry

The differences in mean optical density of TH-positive cells in the substantia nigra among the groups were statistically significant [one-way ANOVA, *F*(3, 20) = 72.71, *P* < 0.01, η^2^*p* = 0.916). *Post-hoc* analysis revealed that the mean optical density in the PD group was significantly lower than that in the control group (0.195 ± 0.056 vs. 1.000 ± 0.145, *P* < 0.01, Cohen’s *d* = 7.33); the mean optical density in the PD group was significantly lower than that in the Gen+PD group (0.195 ± 0.056 vs. 0.435 ± 0.056, *P* < 0.01, Cohen’s *d* = 4.29); the mean optical density in the Acc+PD group was significantly higher than that in the PD group (0.683 ± 0.110 vs. 0.195 ± 0.056, *P* < 0.01, Cohen’s *d* = 5.60); and the mean optical density in the Acc+PD group was significantly higher than that in the Gen+PD group (0.683 ± 0.110 vs. 0.435 ± 0.056, *P* < 0.01, Cohen’s *d* = 2.85) (Figure 3A,B).

**FIGURE 3:**
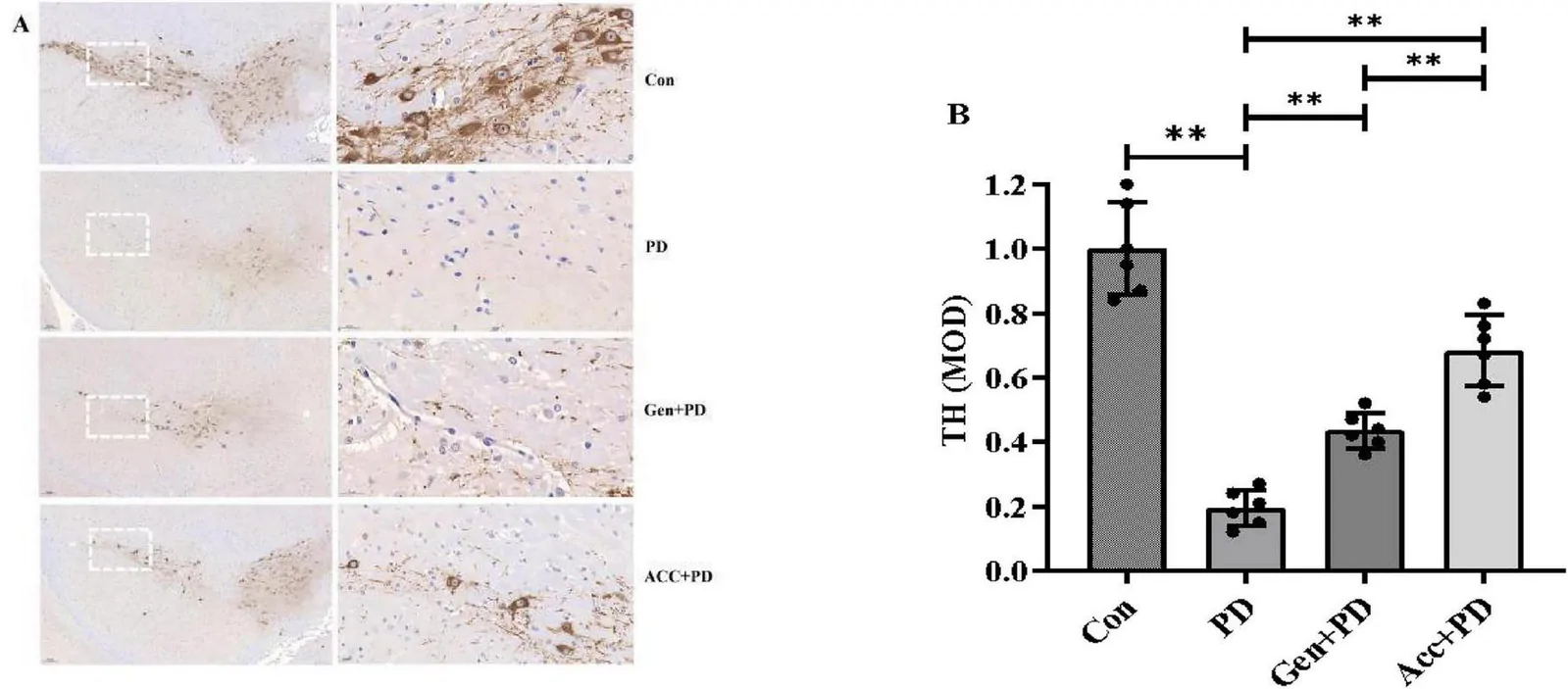
Immunohistochemical results of the substantia nigra. **(A)** Representative images of TH-immunopositive cell staining in the substantia nigra (**A**-Left: × 20; **A**-Right: × 40). **(B)** Mean optical density of TH-immunopositive cell in the striatum. **P < 0.01.

### 3.2 Effects of exercise on balance ability, motor coordination, and spontaneous activity in PD model mice

Balance assessment: The differences in negative geotaxis turning latency among the groups were statistically significant [one-way ANOVA, *F*(3, 44) = 143.78, *P* < 0.01, η^2^*p* = 0.907]. *Post-hoc* analysis revealed that the turning latency in the PD group was significantly longer than that in the control group (15.534 ± 2.621 vs. 2.958 ± 1.140 s, *P* < 0.01, Cohen’s *d* = 6.22); the turning latency in the PD group was significantly longer than that in the Gen+PD group (15.534 ± 2.621 vs. 9.075 ± 0.882 s, *P* < 0.01, Cohen’s *d* = 3.27); the turning latency in the Acc+PD group was significantly shorter than that in the PD group (5.906 ± 0.868 vs. 15.534 ± 2.621 s, *P* < 0.01, Cohen’s *d* = 4.93); and the turning latency in the Acc+PD group was significantly shorter than that in the Gen+PD group (5.906 ± 0.868 vs. 9.075 ± 0.882 s, *P* < 0.01, Cohen’s *d* = 3.62) (Figure 4A).

**FIGURE 4:**
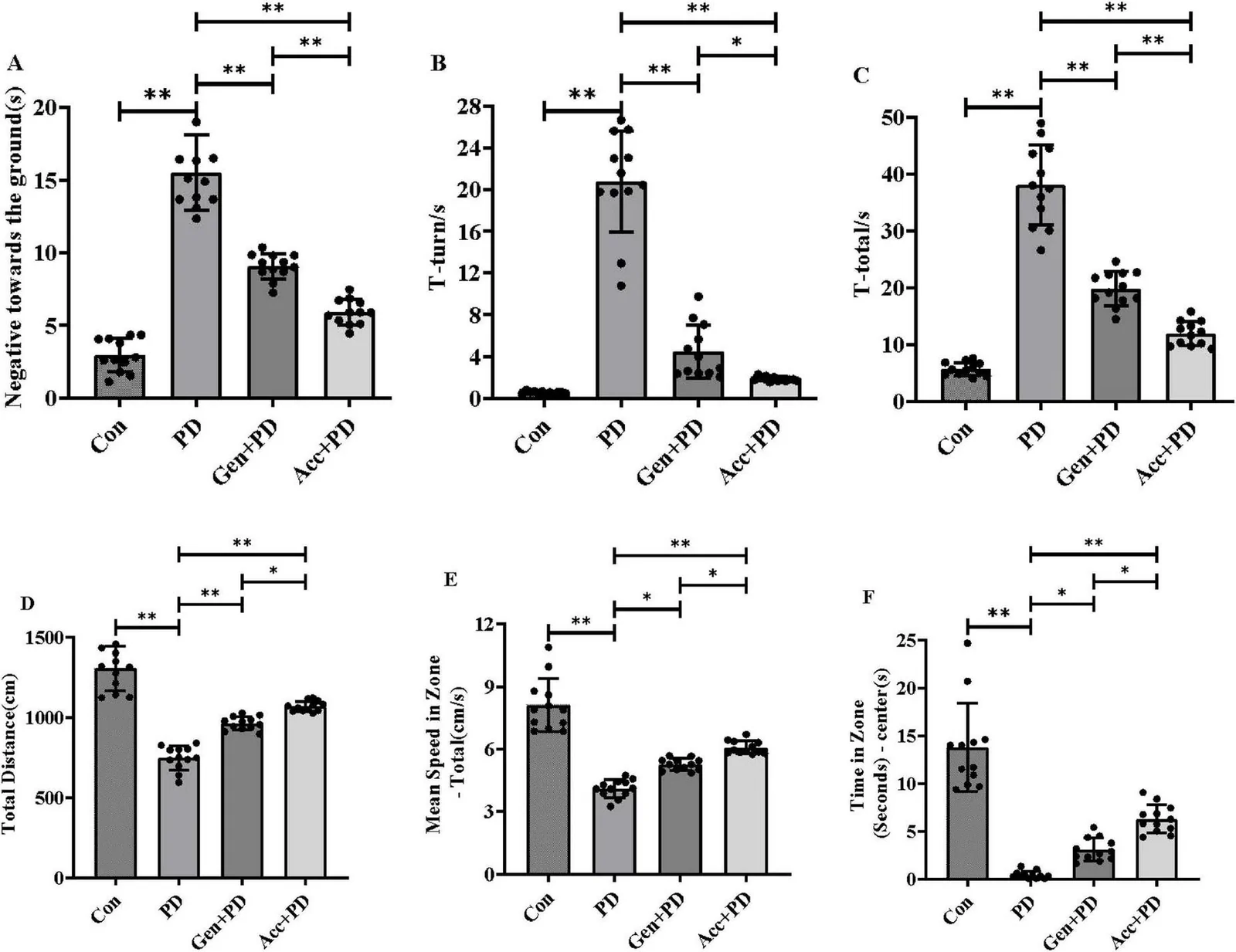
Comparisons of behavioral tests among groups of mice. **(A)** Comparison of the turning latency from head-down to head-up in the negative geotaxis test among groups of mice. **(B)** Comparison of the automatic turning latency in the pole test among groups of mice. **(C)** Comparison of the total pole test time among groups of mice. **(D)** Comparison of the total distance traveled in the open field test among groups of mice. **(E)** Comparison of the mean speed in the open field test among groups of mice. **(F)** Comparison of the time spent in the center zone in the open field test among groups of mice. *P < 0.05, **P < 0.01.

Motor coordination assessment: The differences in T-turn latency in the pole-climbing test among the groups were statistically significant [one-way ANOVA, *F*(3, 44) = 140.75, *P* < 0.01, η^2^*p* = 0.906]. *Post-hoc* analysis revealed that the T-turn latency in the PD group was significantly longer than that in the control group (20.768 ± 4.844 vs. 0.547 ± 0.130 s, *P* < 0.01, Cohen’s *d* = 5.90); the T-turn latency in the PD group was significantly longer than that in the Gen+PD group (20.768 ± 4.844 vs. 4.468 ± 2.550 s, *P* < 0.01, Cohen’s *d* = 4.19); the T-turn latency in the Acc+PD group was significantly shorter than that in the PD group (1.841 ± 0.219 vs. 20.768 ± 4.844 s, *P* < 0.01, Cohen’s *d* = 5.52); and the T-turn latency in the Acc+PD group was significantly shorter than that in the Gen+PD group (1.841 ± 0.219 vs. 4.468 ± 2.550 s, *P* < 0.05, Cohen’s *d* = 1.45) (Figure 4B).

The differences in total pole-climbing time among the groups were statistically significant [one-way ANOVA, *F*(3, 44) = 145.06, *P < 0.01*, η^2^*p* = 0.908]. *Post-hoc* analysis revealed that the total pole-climbing time in the PD group was significantly longer than that in the control group (38.074 ± 7.092 vs. 5.664 ± 1.155 s, *P* < 0.01, Cohen’s *d* = 6.38); the total pole-climbing time in the PD group was significantly longer than that in the Gen+PD group (38.074 ± 7.092 vs. 19.829 ± 3.017 s, *P* < 0.01, Cohen’s *d* = 3.27); the total pole-climbing time in the Acc+PD group was significantly shorter than that in the PD group (11.911 ± 2.150 vs. 38.074 ± 7.092 s, *P* < 0.01, Cohen’s *d* = 4.99); and the total pole-climbing time in the Acc+PD group was significantly shorter than that in the Gen+PD group (11.911 ± 2.150 vs. 19.829 ± 3.017 s, *P* < 0.01, Cohen’s *d* = 3.02) (Figure 4C).

Spontaneous locomotor activity: The differences in total locomotor distance in the open field test among the groups were statistically significant [one-way ANOVA, *F*(3, 44) = 95.39, *P* < 0.01, η^2^*p* = 0.867]. *Post-hoc* analysis revealed that the total locomotor distance in the PD group was significantly shorter than that in the control group (747.941 ± 74.528 vs. 1308.987 ± 138.163 cm, *P* < 0.01, Cohen’s *d* = 5.05); the total locomotor distance in the PD group was significantly shorter than that in the Gen+PD group (747.941 ± 74.528 vs. 964.008 ± 41.105 cm, *P* < 0.01, Cohen’s *d* = 3.50); the total locomotor distance in the Acc+PD group was significantly longer than that in the PD group (1070.413 ± 32.179 vs. 747.941 ± 74.528 cm, *P* < 0.01, Cohen’s *d* = 5.62); and the total locomotor distance in the Acc+PD group was significantly longer than that in the Gen+PD group (1070.413 ± 32.179 vs. 964.008 ± 41.105 cm, *P < 0.05*, Cohen’s d = 2.88).

The differences in mean locomotor velocity in the open field test among the groups were statistically significant [one-way ANOVA, *F*(3, 44) = 69.86, *P* < 0.01, η^2^*p* = 0.826]. *Post-hoc* analysis revealed that the mean velocity in the PD group was significantly lower than that in the control group (4.106 ± 0.426 vs. 8.112 ± 1.266 cm/s, *P* < 0.01, Cohen’s *d* = 4.24); the mean velocity in the PD group was significantly lower than that in the Gen+PD group (4.106 ± 0.426 vs. 5.265 ± 0.279 cm/s, *P* < 0.05, Cohen’s *d* = 3.17); the mean velocity in the Acc+PD group was significantly higher than that in the PD group (6.073 ± 0.309 vs. 4.106 ± 0.426 cm/s, *P* < 0.01, Cohen’s *d* = 5.29); and the mean velocity in the Acc+PD group was significantly higher than that in the Gen+PD group (6.073 ± 0.309 vs. 5.265 ± 0.279 cm/s, *P < 0.05*, Cohen’s d = 2.75) (Figures 4D,E, 5A–D).

**FIGURE 5:**
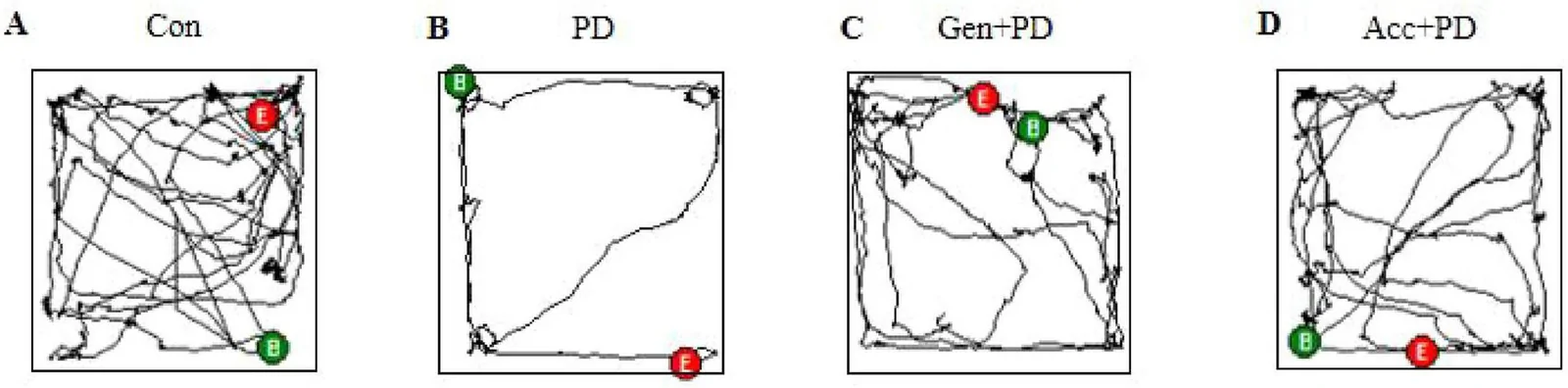
Open field test trajectory plots. **(A)** Open-field test trajectory plot of Con-group mice. **(B)** Open-field test trajectory plot of PD-group mice. **(C)** Open-field test trajectory plot of Gen+PD-group mice. **(D)** Open-field test trajectory plot of Acc+PD-group mice. On trajectory plots, B denotes the starting point (Begin), and E denotes the end point (End).

Anxiety-like behavior: The differences in time spent in the central zone of the open field test among the groups were statistically significant [one-way ANOVA, *F*(3, 44) = 63.16, *P* < 0.01, η^2^*p* = 0.812]. *Post-hoc* analysis revealed that the time spent in the central zone in the PD group was significantly shorter than that in the control group (0.440 ± 0.392 vs. 13.776 ± 4.645 s, *P* < 0.01, Cohen’s *d* = 4.05); the time spent in the central zone in the PD group was significantly shorter than that in the Gen+PD group (0.440 ± 0.392 vs. 3.088 ± 1.187 s, *P* < 0.05, Cohen’s *d* = 2.97); the time spent in the central zone in the Acc+PD group was significantly longer than that in the PD group (6.288 ± 1.474 vs. 0.440 ± 0.392 s, *P* < 0.01, Cohen’s *d* = 5.43); and the time spent in the central zone in the Acc+PD group was significantly longer than that in the Gen+PD group (6.288 ± 1.474 vs. 3.088 ± 1.187 s, *P* < 0.05, Cohen’s *d* = 2.39) (Figures 4F, 5A–D).

Taken together, the accelerated/challenging exercise preconditioning group (Acc+PD) demonstrated consistently superior performance across all behavioral endpoints—encompassing balance, motor coordination, spontaneous locomotion, and anxiety-like behavior—compared to both the PD sedentary group and the conventional exercise group (Gen+PD). This consistent pattern indicates that exercise paradigms incorporating progressive task complexity confer greater functional neuroprotection than constant-speed training in the 6-OHDA model of PD.

### 3.3 Comparison of striatal DA content among groups

The differences in striatal DA content among the groups were statistically significant [one-way ANOVA, *F*(3, 20) = 9.33, *P < 0.01*, η^2^*p* = 0.583]. *Post-hoc* analysis revealed that the DA content in the PD group was significantly lower than that in the control group (16.497 ± 1.487 vs. 21.366 ± 1.711 ng/mg, *P* < 0.01, Cohen’s *d* = 3.04); the DA content in the PD group was significantly lower than that in the Gen+PD group (16.497 ± 1.487 vs. 18.665 ± 1.966 ng/mg, *P* < 0.05, Cohen’s *d* = 1.23); the DA content in the Acc+PD group was significantly higher than that in the PD group (20.787 ± 1.739 vs. 16.497 ± 1.487 ng/mg, *P* < 0.01, Cohen’s *d* = 2.65); and the DA content in the Acc+PD group was significantly higher than that in the Gen+PD group (20.787 ± 1.739 vs. 18.665 ± 1.966 ng/mg, *P* < 0.05, Cohen’s *d* = 1.14) (Figure 6A).

**FIGURE 6:**
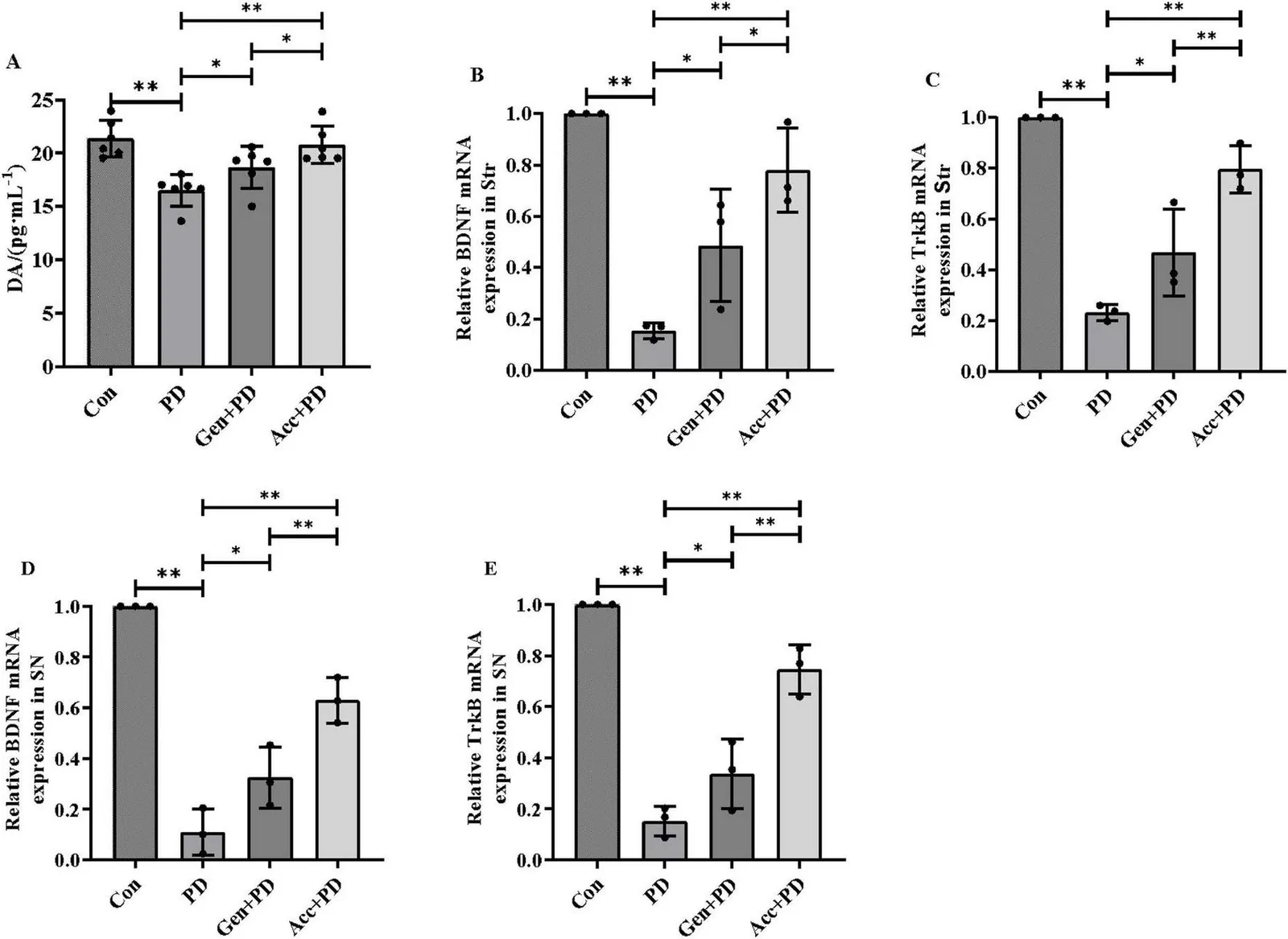
Comparisons of striatal DA concentration and BDNF, TrkB mRNA expression levels in the striatum and substantia nigra among groups of mice. **(A)** Comparison of striatal DA concentration among groups of mice. **(B)** Comparison of BDNF mRNA expression levels in the striatum among groups of mice. **(C)** Comparison of TrkB mRNA expression levels in the striatum among groups of mice. **(D)** Comparison of BDNF mRNA expression levels in the substantia nigra among groups of mice. **(E)** Comparison of TrkB mRNA expression levels in the substantia nigra among groups of mice. *P < 0.05, **P < 0.01.

### 3.4 Comparison of BDNF and TrkB mRNA expression levels in the striatum and substantia nigra among groups

The differences in relative expression levels of striatal BDNF mRNA among the groups were statistically significant [one-way ANOVA, *F*(3, 8) = 19.78, *P* < 0.01, η^2^*p* = 0.881]. *Post-hoc* analysis revealed that the relative expression level of striatal BDNF mRNA in the PD group was significantly lower than that in the control group (0.188 ± 0.029 vs. 1.000 ± 0.000, *P* < 0.01, Cohen’s *d* = 39.60); the relative expression level in the Gen+EX group was significantly higher than that in the PD group (0.486 ± 0.218 vs. 0.188 ± 0.029, *P* < 0.05, Cohen’s *d* = 1.92); the relative expression level in the Acc+EX group was significantly higher than that in the PD group (0.780 ± 0.164 vs. 0.188 ± 0.029, *P* < 0.01, Cohen’s *d* = 5.03); and the relative expression level in the Acc+EX group was significantly higher than that in the Gen+EX group (0.780 ± 0.164 vs. 0.486 ± 0.218, *P* < 0.05, Cohen’s *d* = 1.52) (Figure 6B).

The differences in relative expression levels of striatal TrkB mRNA among the groups were statistically significant [one-way ANOVA, *F*(3, 8) = 35.57, *P* < 0.01, η^2^*p* = 0.930]. *Post-hoc* analysis revealed that the relative expression level of striatal TrkB mRNA in the PD group was significantly lower than that in the control group (0.232 ± 0.031 vs. 1.000 ± 0.000, *P* < 0.01, Cohen’s *d* = 35.04); the relative expression level in the Gen+EX group was significantly higher than that in the PD group (0.469 ± 0.173 vs. 0.232 ± 0.031, *P* < 0.05, Cohen’s *d* = 1.91); the relative expression level in the Acc+EX group was significantly higher than that in the PD group (0.797 ± 0.092 vs. 0.232 ± 0.031, *P* < 0.01, Cohen’s *d* = 8.23); and the relative expression level in the Acc+EX group was significantly higher than that in the Gen+EX group (0.797 ± 0.092 vs. 0.469 ± 0.173, *P* < 0.01, Cohen’s *d* = 2.37) (Figure 6C).

The differences in relative expression levels of substantia nigra BDNF mRNA among the groups were statistically significant [one-way ANOVA, *F*(3, 8) = 58.891, *P* < 0.01, η^2^*p* = 0.957]. *Post-hoc* analysis revealed that the relative expression level of substantia nigra BDNF mRNA in the PD group was significantly lower than that in the control group (0.110 ± 0.091 vs. 1.000 ± 0.000, *P* < 0.01, Cohen’s *d* = 13.83); the relative expression level in the Gen+EX group was significantly higher than that in the PD group (0.325 ± 0.120 vs. 0.110 ± 0.091, *P* < 0.05, Cohen’s *d* = 2.02); the relative expression level in the Acc+EX group was significantly higher than that in the PD group (0.630 ± 0.089 vs. 0.110 ± 0.091, *P* < 0.01, Cohen’s *d* = 5.78); and the relative expression level in the Acc+EX group was significantly higher than that in the Gen+EX group (0.630 ± 0.089 vs. 0.325 ± 0.120, *P* < 0.01, Cohen’s *d* = 2.89) (Figure 6D).

The differences in relative expression levels of substantia nigra TrkB mRNA among the groups were statistically significant [one-way ANOVA, *F*(3, 8) = 65.98, *P* < 0.01, η^2^*p* = 0.961]. *Post-hoc* analysis revealed that the relative expression level of substantia nigra TrkB mRNA in the PD group was significantly lower than that in the control group (0.153 ± 0.059 vs. 1.000 ± 0.000, *P* < 0.01, Cohen’s *d* = 20.30); the relative expression level in the Gen+EX group showed no significant difference compared with the PD group (0.337 ± 0.135 vs. 0.153 ± 0.059, *P* < 0.05); the relative expression level in the Acc+EX group was significantly higher than that in the PD group (0.746 ± 0.097 vs. 0.153 ± 0.059, *P* < 0.01, Cohen’s *d* = 7.39); and the relative expression level in the Acc+EX group was significantly higher than that in the Gen+EX group (0.746 ± 0.097 vs. 0.337 ± 0.135, *P* < 0.01, Cohen’s *d* = 3.48) (Figure 6E).

### 3.5 Comparison of TH, BDNF, and TrkB protein expression in the substantia nigra of mice

The differences in relative expression levels of BDNF protein among the groups were statistically significant [one-way ANOVA, *F*(3, 8) = 23.488, *P* < 0.01, η^2^*p* = 0.898]. *Post-hoc* analysis revealed that the relative expression level of BDNF protein in the PD group was significantly lower than that in the control group (0.394 ± 0.059 vs. 1.000 ± 0.121, *P* < 0.01, Cohen’s *d* = 6.37); the relative expression level in the Gen+EX group was significantly higher than that in the PD group (0.644 ± 0.077 vs. 0.394 ± 0.059, *P* < 0.05, Cohen’s *d* = 3.64); the relative expression level in the Acc+EX group was significantly higher than that in the PD group (0.868 ± 0.110 vs. 0.394 ± 0.059, *P* < 0.01, Cohen’s *d* = 5.37); and the relative expression level in the Acc+EX group was significantly higher than that in the Gen+EX group (0.868 ± 0.110 vs. 0.644 ± 0.077, *P* < 0.05, Cohen’s *d* = 2.36) (Figure 7A,B).

**FIGURE 7:**
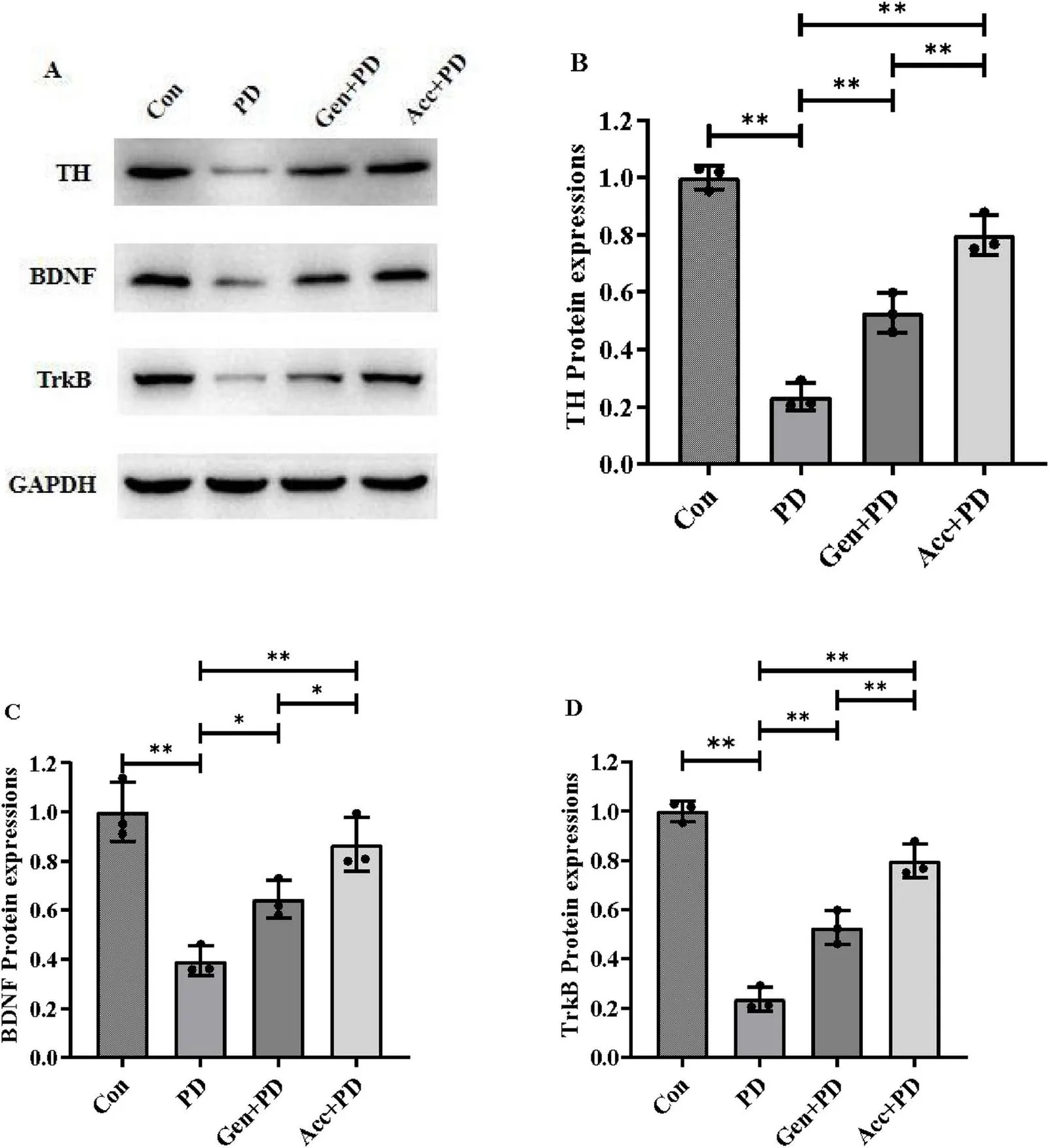
Comparisons of TH, BDNF, and TrkB expression levels in the substantia nigra among groups of mice. **(A)** Immunoblot images of TH protein expression in the substantia nigra among groups of mice. **(B)** Comparison of TH expression levels in the substantia nigra among groups of mice. **(C)** Comparison of BDNF expression levels in the substantia nigra among groups of mice. **(D)** Comparison of TrkB expression levels in the substantia nigra among groups of mice. *P < 0.05, **P < 0.01.

The differences in the relative expression levels of TrkB protein among the groups were statistically significant [one-way ANOVA, *F*(3, 8) = 97.747, *P* < 0.01, η^2^*p* = 0.973]. *Post-hoc* LSD analysis revealed that the relative expression level of TrkB protein in the PD group was significantly lower than that in the control group (0.237 ± 0.048 vs. 1.000 ± 0.042, *P* < 0.01, Cohen’s *d* = 16.92); the relative expression level in the Gen+EX group was significantly higher than that in the PD group (0.528 ± 0.069 vs. 0.237 ± 0.048, *P* < 0.01, Cohen’s *d* = 4.90); the relative expression level in the Acc+EX group was significantly higher than that in the PD group (0.799 ± 0.069 vs. 0.237 ± 0.048, *P* < 0.01, Cohen’s *d* = 9.46); and the relative expression level in the Acc+EX group was significantly higher than that in the Gen+EX group (0.799 ± 0.069 vs. 0.528 ± 0.069, *P* < 0.01, Cohen’s *d* = 3.93) (Figure 7A,C).

The differences in the relative expression levels of TH protein among the groups were statistically significant [one-way ANOVA, *F*(3, 8) = 65.512, *P* < 0.01, η^2^*p* = 0.961]. *Post-hoc* LSD analysis revealed that the relative expression level of TH protein in the PD group was significantly lower than that in the control group (0.257 ± 0.073 vs. 1.000 ± 0.057, *P* < 0.01, Cohen’s *d* = 11.35); the relative expression level in the Gen+EX group was significantly higher than that in the PD group (0.541 ± 0.027 vs. 0.257 ± 0.073, *P* < 0.01, Cohen’s *d* = 5.16); the relative expression level in the Acc+EX group was significantly higher than that in the PD group (0.791 ± 0.097 vs. 0.257 ± 0.073, *P* < 0.01, Cohen’s *d* = 6.22); and the relative expression level in the Acc+EX group was significantly higher than that in the Gen+EX group (0.791 ± 0.097 vs. 0.541 ± 0.027, *P* < 0.01, Cohen’s *d* = 3.51) (Figure 7A,D).

## 4 Discussion

Research has demonstrated that to maximize the effects of training, a high level of challenge should be maintained throughout the intervention period (Pavlikova et al., 2020). Conradsson et al. developed a highly challenging and progressive group balance training protocol specifically for PD symptoms (Conradsson et al., 2014). This protocol incorporates principles of motor learning specificity (i.e., exercises targeting motor, sensory, and cognitive impairments in Parkinson’s disease) and progressive overload (i.e., highly demanding balance exercises with a structured temporal progression). The feasibility of this program was evaluated in elderly patients with mild-to-moderate idiopathic PD. The results indicated that 12 weeks of high-challenge progressive balance training (3 sessions/week, 45 min/session) led to significant improvements in balance function in these patients. Furthermore, high attendance rates were maintained throughout the training period, with a low incidence of adverse events (one fall and one episode of hypotension-related vertigo). Consequently, this highly challenging and progressive group balance training regimen was deemed feasible and promising. Conradsson et al. further evaluated the short-term effects of a high-challenge balance training program—which combined dual-tasking with PD-specific balance components—in elderly patients with mild-to-moderate PD (Conradsson et al., 2015). Their findings showed significant improvements in balance and gait performance in the training group compared to the control group. Additionally, although no significant intergroup differences were observed in gait performance during dual-tasking, the training group demonstrated improved performance in executing cognitive tasks while walking. The training group also achieved more favorable outcomes regarding physical activity levels and activities of daily living (ADL). Similarly, Sparrow et al. reported that a 3-month theory-based, highly challenging, and progressive exercise program was effective in reducing falls, improving balance, and decreasing the fear of falling in patients with mild-to-moderate PD (Sparrow et al., 2016). Wallén et al. showed that 10 weeks of high-challenge balance training significantly improved balance control and gait speed in elderly individuals with mild-to-moderate PD (Hoehn and Yahr stages 2–3) (Wallén et al., 2018); however, these training effects diminished after 6 months. In a similar vein, Rennie et al. demonstrated that highly challenging balance and gait training improved gait velocity, rhythm, and variability in PD patients in the short term compared to a usual care group, though no training effects were retained at the 6-month follow-up (Rennie et al., 2021). A meta-analysis by Malmir and Ashrafganjooie (2023) further indicated that high-challenge training had a significant impact on physical function, with significant improvements in Mini-Balance Evaluation Systems Test (Mini-BESTest) scores and walking speed (Malmir and Ashrafganjooie, 2023).

While these clinical studies have established the functional benefits of high-challenge training in PD patients, the neurobiological mechanisms underlying these benefits remain insufficiently characterized. Translating this clinical concept into a preclinical setting requires well-controlled animal models that can isolate the specific contribution of task demand intensity—as distinct from aerobic workload or total exercise volume—to neuroprotective outcomes. The majority of preclinical studies examining exercise-mediated neuroprotection in toxin-based PD models have employed constant-speed treadmill or voluntary wheel-running protocols (Tajiri et al., 2010; Petzinger et al., 2013; Toy et al., 2014). Although these studies have convincingly demonstrated that aerobic exercise preserves TH-positive neurons, restores striatal DA, and upregulates neurotrophic factors, they have not systematically varied the complexity or challenge level of the motor task itself. As a result, the relative contribution of task complexity to neuroprotective outcomes has remained unclear. The present study directly addresses this gap by comparing two rotarod protocols that are matched in modality and session duration but differ specifically in task demand: a constant-speed protocol (Gen) representing conventional training versus an accelerating protocol (Acc) representing challenging training. The results of the present study demonstrated that, compared to the PD group, mice in the Acc+PD group exhibited significantly reduced turn delay, turn latency (T-turn), and total pole-climbing time (T-total). Conversely, total distance traveled, mean velocity, and time spent in the center zone were significantly increased. Furthermore, striatal dopamine (DA) content was significantly higher, and the expression levels of tyrosine hydroxylase (TH) protein in the substantia nigra were significantly upregulated in the Acc+PD group, with these outcomes generally outperforming those of the Gen+PD group. These findings suggest that challenging exercise preconditioning can significantly delay the decline of balance function, spontaneous activity, and motor coordination in PD model mice.

Importantly, a consistent gradient of neuroprotective outcomes was observed across all levels of analysis: while conventional constant-speed rotarod preconditioning (Gen+PD) significantly attenuated PD-related deficits relative to sedentary controls, the accelerated/challenging rotarod protocol (Acc+PD) produced superior outcomes across every behavioral, neurochemical, and molecular endpoint examined. This consistent dose-response pattern suggests that the critical variable associated with neuroprotective efficacy is not merely the presence or absence of physical activity, but rather the degree of “task demand intensity” embedded within the exercise paradigm. We propose that the progressive sensorimotor challenge inherent in accelerating rotarod training—requiring mice to continuously update motor strategies, recruit cerebellar-basal ganglia-cortical circuits, and maintain dynamic balance under escalating difficulty—may engage broader and more functionally relevant neural networks than constant-speed training. This sustained adaptive engagement may more potently stimulate activity-dependent neurotrophic signaling and thereby amplify downstream neuroprotective cascades. The finding that the Acc group consistently outperformed the Gen group—despite engaging in the same type of exercise for the same duration—provides correlative evidence suggesting that progressive task complexity is an independent contributor to exercise-induced neuroprotection, extending the clinical observations of Allen et al. (2011) and Petzinger et al. (2013) into a mechanistically informed preclinical framework. It should be noted, however, that the current experimental design establishes an association rather than definitive causality between task demand intensity and neuroprotective outcomes; direct manipulation of specific signaling nodes will be required to confirm causal relationships. The mechanistic interpretation of the present findings can be organized along a primary axis directly supported by our experimental data, complemented by secondary pathways that provide supportive context from the broader literature.

BDNF and its receptor, TrkB, are widely distributed across multiple brain regions and play critical roles in physiological processes such as neurogenesis, neuronal maturation and maintenance, synaptic plasticity, and neuroprotection. Both the activation and dysfunction of the BDNF-TrkB signaling pathway are closely associated with neurodegeneration in Parkinson’s disease (PD). In the substantia nigra (SN) of PD patients, the expression of BDNF and TrkB is significantly reduced. Furthermore, the loss of TrkB receptors leads to the degeneration of dopaminergic (DAergic) neurons and the aggregation of alpha-synuclein (α-Syn) in the SN. Animal model studies have demonstrated that both glial cell-derived neurotrophic factor (GDNF) and BDNF can alleviate the loss of nigrostriatal DAergic neurons induced by injury. Expressing BDNF in the striatum or midbrain has been shown to reduce 6-OHDA-induced nigrostriatal neuronal loss and improve behavioral performance; additionally, BDNF can prevent the neurotoxicity of MPTP or 6-OHDA in DAergic neurons of rats (Tajiri et al., 2010; Sathiya et al., 2013; Simon et al., 2020; Kang et al., 2022). Targeting the BDNF/TrkB signaling pathway with specific activators in MPTP-induced human SNCA transgenic PD mice significantly reduced α-Syn N103 fragments in the SN, markedly increased the number of tyrosine hydroxylase (TH)-positive DAergic neurons in the SN and striatum, significantly elevated striatal DA content, and substantially improved motor coordination and balance (Kang et al., 2022). Experimental animal studies indicate that various types or intensities of exercise can significantly upregulate brain levels of BDNF and TrkB proteins, mediating the positive effects of physical activity on brain function and neural network plasticity through a TrkB-dependent mechanism (Lin et al., 2012; Shimoda et al., 2024). However, there is currently no literature reporting which specific type or intensity of exercise provides more potent activation of BDNF/TrkB signaling. Our findings demonstrate that in PD mice, the expression levels of TH, BDNF, and TrkB mRNA in the SN, as well as BDNF and TrkB protein levels in the striatum, were downregulated, accompanied by a significant reduction in striatal DA concentration. Notably, 4-week pre-training with both regular rotarod and challenging rotarod significantly upregulated the expression of TH, BDNF, and TrkB mRNA in the SN and BDNF/TrkB protein in the striatum of PD mice. However, challenging rotarod pre-training proved superior to regular rotarod pre-training, providing correlative preclinical evidence suggesting that progressive task complexity may be an important factor associated with enhanced BDNF/TrkB signaling, and is consistent with the interpretation that challenging exercise exerts a more robust neuroprotective effect through enhanced activity-dependent neurotrophic signaling. Nonetheless, it must be acknowledged that the present study did not include direct pathway manipulation—such as pharmacological TrkB antagonism or conditional BDNF knockdown—and therefore the observed upregulation of BDNF/TrkB should be interpreted as strongly associated with, rather than causally established as mediating, the neuroprotective effects of challenging exercise preconditioning.

Beyond the primary BDNF/TrkB/ TH axis examined in this study, prior literature suggests that several additional signaling cascades may contribute to exercise-mediated neuroprotection and warrant consideration as potential downstream or parallel mechanisms. CREB (cAMP response element-binding protein), a well-characterized transcription factor downstream of TrkB activation, regulates the transcription of multiple pro-survival genes including BDNF itself, forming a positive feedback loop that may sustain neurotrophic support (Jang et al., 2017; Wu et al., 2011). The PI3K/Akt and MAPK/ERK pathways, which are canonical intracellular effectors of TrkB receptor activation, have been implicated in promoting neuronal survival, inhibiting pro-apoptotic signaling, and enhancing synaptic plasticity in various PD models (Simon et al., 2020). Additionally, GDNF has been shown to act synergistically with BDNF in supporting nigrostriatal dopaminergic neuron survival (Tajiri et al., 2010; Sathiya et al., 2013). Although these pathways were not directly measured in the present study, they represent plausible downstream mediators of the BDNF/TrkB signaling upregulation observed here and constitute important targets for future mechanistic investigation. The present study employed the 6-OHDA model, which remains one of the most widely used and well-characterized toxin-based paradigms for studying dopaminergic neurodegeneration and evaluating neuroprotective interventions in PD research. It is therefore important to situate our findings within the growing body of literature examining exercise-based neuroprotection specifically in this model system. Previous studies using the 6-OHDA model have demonstrated that various forms of exercise—including forced treadmill running (Tajiri et al., 2010; Toy et al., 2014), voluntary wheel running, and skilled motor training—can attenuate dopaminergic neuron loss, restore striatal DA levels, upregulate neurotrophic factor expression, and improve motor behavioral outcomes. Our findings are consistent with and extend this literature by demonstrating that, within a single exercise modality (rotarod), the degree of task challenge is a critical variable that determines the magnitude of neuroprotective benefit—an aspect that has not been systematically addressed in prior 6-OHDA exercise studies.

Importantly, the mechanistic landscape of exercise-mediated neuroprotection in 6-OHDA models is not limited to the neuronal BDNF/TrkB axis examined in the present study. Recent work by Freire et al. (2025) has highlighted the significance of glial and apoptotic responses in modulating nigrostriatal vulnerability and functional outcomes following 6-OHDA lesioning. Specifically, glial cells—including astrocytes and microglia—play dual roles in neurodegenerative processes: reactive astrogliosis and microglial activation can exacerbate neuroinflammation and oxidative damage, while appropriately regulated glial responses contribute to neurotrophic support, synaptic homeostasis, and debris clearance. Exercise has been shown to shift glial phenotypes toward neuroprotective states, reducing pro-inflammatory cytokine release and enhancing anti-inflammatory and trophic functions. Similarly, the balance between pro-apoptotic (e.g., Bax, Caspase-3) and anti-apoptotic (e.g., Bcl-2) signaling—which we did not directly assess but is well-documented in the 6-OHDA context—represents another critical determinant of dopaminergic neuron survival that may be differentially modulated by varying levels of exercise challenge. Integrating these perspectives with our findings, we propose that the superior neuroprotective outcomes observed in the Acc group may reflect not only enhanced neuronal BDNF/TrkB signaling but also more favorable modulation of non-neuronal contributors—including glial reactivity and apoptotic balance—although this hypothesis remains to be tested directly. Future studies should incorporate assessments of glial activation markers (e.g., GFAP, Iba-1), neuroinflammatory cytokine profiles, and apoptotic pathway components alongside neurotrophic signaling to provide a more comprehensive mechanistic characterization of how exercise challenge intensity influences the nigrostriatal neuroprotective response in 6-OHDA models.

In summary, challenging exercise has demonstrated significant short-term efficacy in alleviating behavioral dysfunction associated with Parkinson’s Disease (PD); however, further investigation is required regarding the maintenance of its long-term effects. In clinical practice, personalized challenging training protocols should be tailored to individual patient differences, incorporating appropriate safety measures to ensure both efficacy and participant safety. Future research should further elucidate the underlying mechanisms of challenging training and explore the design of continuous, progressive training programs to ensure sustained long-term benefits for patients. From a translational perspective, the present findings carry important implications for how “exercise challenge intensity” may inform future exercise prescription strategies in PD, which can be considered along three interrelated dimensions: dose, complexity, and progression.

First, regarding dose, our data suggest that the neuroprotective response is not simply a function of total exercise volume or duration. Both the Gen and Acc groups trained for the same number of sessions and the same duration per session, yet the Acc group demonstrated consistently superior outcomes. This indicates that the qualitative characteristics of the exercise stimulus—specifically, the level of motor challenge imposed per unit of training time—may be a more critical dosing parameter than cumulative exercise quantity alone. In preclinical research, this implies that future studies should systematically report not only treadmill speed or rotarod duration but also explicitly characterize the adaptive demand profile of the exercise protocol (e.g., fixed vs. accelerating, predictable vs. variable). Second, regarding complexity, our accelerating rotarod paradigm required continuous motor recalibration, dynamic postural adjustment, and real-time sensorimotor integration under escalating difficulty—demands that are qualitatively absent in constant-speed protocols. This distinction mirrors the clinical concept of “skill-based” versus “aerobic-only” exercise interventions (Petzinger et al., 2013). Clinically, this supports the incorporation of task complexity as a deliberate prescription parameter: rehabilitation programs should move beyond simple aerobic targets (e.g., heart rate zones, metabolic equivalents) to also specify the motor-cognitive challenge level of prescribed activities. Rehabilitation programs should not only ensure adequate exercise volume and intensity but should also deliberately incorporate progressive motor challenges—such as variable-speed training, dual-task paradigms, or perturbation-based balance exercises—to maximize neuroprotective and functional outcomes. Third, regarding progression, the clinical literature reviewed in this study consistently demonstrates that training effects of high-challenge balance programs diminish after cessation (Wallén et al., 2018; Rennie et al., 2021), suggesting that static training protocols—even if initially challenging—may lose their neuroprotective potency as adaptation occurs. Our preclinical model, in which the rotarod accelerated progressively within each trial, provides a built-in mechanism of continuous challenge escalation. Translating this principle clinically implies that exercise prescriptions for PD should incorporate structured, periodic increases in task difficulty—analogous to the progressive overload principle in strength training—to maintain the adaptive stimulus above the threshold required for sustained neuroplastic benefit. Specific strategies may include scheduled increments in speed variability, introduction of novel motor sequences, addition of cognitive dual-task loads, or progression from stable to unstable support surfaces. Taken together, these three dimensions—dose (challenge per unit time rather than total volume), complexity (motor-cognitive demand level), and progression (structured escalation of difficulty)—constitute a preliminary framework for “challenge-based exercise dosing” that may guide both preclinical protocol design and clinical rehabilitation programming in PD. However, the optimal parameters within each dimension remain to be defined through systematic dose-finding studies in both animal models and patient populations.

Several noteworthy limitations of this study should be acknowledged. First, the time interval between pretreatment, modeling, and behavioral assessment was relatively short, and the study lacked long-term longitudinal follow-up after modeling. Second, while this study focused on the BDNF-TrkB-TH axis and confirmed changes in upstream molecules (BDNF, TrkB, and TH) at both mRNA and protein levels, it lacked an in-depth analysis of downstream regulatory details (such as the activation state of CREB and its precise transcriptional regulation of TH). Furthermore, other core pathways closely related to neuroprotection, such as pro-survival/ anti-apoptotic pathways (e.g., PI3K/Akt and MAPK/ERK), were not extensively explored. Most importantly, the study lacks functional validation experiments—such as the application of specific TrkB antagonists or the use of conditional BDNF knockdown/knockout models—to rigorously establish the necessity and causality of BDNF-TrkB signaling in mediating the protective effects of exercise pretreatment. Future research should integrate long-term follow-up to clarify the durability of these protective effects and provide a deeper analysis of the downstream CREB/PI3K/MAPK pathways. Causal mechanisms should be verified using pharmacological antagonists or gene-editing techniques. Additionally, randomized controlled trials (RCTs) are needed to compare the preventive and disease-modifying effects of “challenging aerobic exercise” versus “conventional aerobic exercise” in high-risk PD populations or early-stage patients, ultimately establishing objective intensity-dosage guidelines.

## 5 Conclusion

Four weeks of challenging rotarod exercise pretreatment was significantly superior to conventional rotarod exercise pretreatment in delaying behavioral dysfunction, increasing striatal dopamine (DA) concentration, and upregulating the expression of TH, BDNF, and TrkB proteins in the substantia nigra, as well as BDNF and TrkB mRNA levels in the striatum of PD model mice. These findings indicate that challenging rotarod exercise pretreatment exerts a more potent neuroprotective effect.

## Data Availability

The raw data supporting the conclusions of this article will be made available by the authors, without undue reservation.
